# Supplementary material for: Impact of Fertilizer on Crop Yield and C:N:P Stoichiometry in Arid and Semi-Arid Soil
Source: Int J Environ Res Public Health. 2021 Apr 20;18(8):4341. doi: 10.3390/ijerph18084341 (PMC8072689; doi:10.3390/ijerph18084341)
Supplement: Supplementary file 1 [file ijerph-18-04341-s001.zip › ijerph-1160077-supplementary.pdf]

Table S1. Basic physical and chemical properties of the soil before fertilization.

**Fertilization treatments**	**SOC (g/kg)**	**TN(g/kg)**	**TP(g/kg)**	**AN(mg/kg)**	**AP(mg/kg)**	**pH**
**CK**	2.68	0.32	0.53	26.40	2.28	8.47
**O**	4.73	0.51	0.58	38.20	9.08	8.45
**ON**	4.24	0.52	0.59	44.40	5.14	8.41
**ONP**	4.27	0.49	0.74	33.70	32.89	8.43
**OP**	4.64	0.54	0.77	36.50	42.06	8.38
**NK**	2.84	0.36	0.52	28.50	1.11	8.45
**NP**	3.37	0.41	0.72	35.40	19.30	8.44
**NPK**	3.21	0.37	0.66	29.20	20.38	8.38
**PK**	2.77	0.32	0.70	24.00	19.02	8.47

Notes: CK: no fertilization; O, 0.75 kg/m^2 organic fertilizer; ON, 0.75 kg/m^2 organic fertilizer and 0.021kg/m^2 nitrogen; ONP, 0.75 kg/m^2 organic fertilizer, 0.021kg/m^2 nitrogen and 0.017 kg/m^2 phosphorus; OP, 0.75 kg/m^2 organic fertilizer and 0.017 kg/m^2 phosphorus; NP, 0.017 kg/m^2 phosphorus and 0.021kg/m^2 nitrogen; NK, 0.012 kg/m^2 potash and 0.021kg/m^2 nitrogen; NPK, 0.012 kg/m^2 potash, 0.021kg/m^2 nitrogen and 0.017 kg/m^2 phosphorus; PK, 0.012 kg/m^2 potash and 0.017 kg/m^2 phosphorus.

Table S2. The slope parameters of the linear regression models for soil organic carbon (SOC) content.

Fertilization treatments	Equations	R^2	P
**CK**	Y=0.12X-240.32	0.886	0.002
**O**	Y=0.24X-477.02	0.825	0.005
**ON**	Y=0.23X-464.51	0.871	0.002
**ONP**	Y=0.15X-303.15	0.381	N
**OP**	Y=0.21X-411.61	0.706	0.018
**NK**	Y=0.12X-228.61	0.839	0.004
**NP**	Y=0.13X-253.18	0.934	0.000
**NPK**	Y=0.12X-239.15	0.899	0.001
**PK**	Y=0.13X-259.64	0.963	0.000

Notes: CK: no fertilization; O, 0.75 kg/m^2 organic fertilizer; ON, 0.75 kg/m^2 organic fertilizer and 0.021kg/m^2 nitrogen; ONP, 0.75 kg/m^2 organic fertilizer, 0.021kg/m^2 nitrogen and 0.017 kg/m^2 phosphorus; OP, 0.75 kg/m^2 organic fertilizer and 0.017 kg/m^2 phosphorus; NP, 0.017 kg/m^2 phosphorus and 0.021kg/m^2 nitrogen; NK, 0.012 kg/m^2 potash and 0.021kg/m^2 nitrogen; NPK, 0.012 kg/m^2 potash, 0.021kg/m^2 nitrogen and 0.017 kg/m^2 phosphorus; PK, 0.012 kg/m^2 potash and 0.017 kg/m^2 phosphorus.

Table S3. The slope parameters of the linear regression models for soil total nitrogen (TN) content.

**Fertilization treatments**	**Equations**	**R^2**	**P**
**CK**	Y=0.006X-12.580	0.734	0.014
**O**	Y=0.017X-32.632	0.846	0.003
**ON**	Y=0.017X-33.550	0.821	0.005
**ONP**	Y=0.010X-20.195	0.206	N
**OP**	Y=0.012X-23.470	0.491	N
**NK**	Y=0.005X-10.448	0.568	N
**NP**	Y=0.006X-11.656	0.472	N
**NPK**	Y=0.005X-9.008	0.627	0.034
**PK**	Y=0.005X-10.089	0.747	0.012

Notes: CK: no fertilization; O, 0.75 kg/m^2 organic fertilizer; ON, 0.75 kg/m^2 organic fertilizer and 0.021kg/m^2 nitrogen; ONP, 0.75 kg/m^2 organic fertilizer, 0.021kg/m^2 nitrogen and 0.017 kg/m^2 phosphorus; OP, 0.75 kg/m^2 organic fertilizer and 0.017 kg/m^2 phosphorus; NP, 0.017 kg/m^2 phosphorus and 0.021kg/m^2 nitrogen; NK, 0.012 kg/m^2 potash and 0.021kg/m^2 nitrogen; NPK, 0.012 kg/m^2 potash, 0.021kg/m^2 nitrogen and 0.017 kg/m^2 phosphorus; PK, 0.012 kg/m^2 potash and 0.017 kg/m^2 phosphorus.

Table S4. The slope parameters of the linear regression models for soil total phosphorus (TP) content.

**Fertilization treatments**	**Equations**	**R2**	**P**
**CK**	Y=0.005X-8.544	0.283	N
**O**	Y=0.003X-6.171	0.161	N
**ON**	Y=-0.001X+2.917	0.060	N
**ONP**	Y=0.009X-17.891	0.791	0.007
**OP**	Y=0.007X-12.418	0.576	0.048
**NK**	Y=0.000X-0.375	0.006	N
**NP**	Y=0.006X-10.876	0.400	N
**NPK**	Y=0.009X-17.323	0.825	0.005
**PK**	Y=0.008X-14.477	0.743	0.013

Notes: CK: no fertilization; O, 0.75 kg/m^2 organic fertilizer; ON, 0.75 kg/m^2 organic fertilizer and 0.021kg/m^2 nitrogen; ONP, 0.75 kg/m^2 organic fertilizer, 0.021kg/m^2 nitrogen and 0.017 kg/m^2 phosphorus; OP, 0.75 kg/m^2 organic fertilizer and 0.017 kg/m^2 phosphorus; NP, 0.017 kg/m^2 phosphorus and 0.021kg/m^2 nitrogen; NK, 0.012 kg/m^2 potash and 0.021kg/m^2 nitrogen; NPK, 0.012 kg/m^2 potash, 0.021kg/m^2 nitrogen and 0.017 kg/m^2 phosphorus; PK, 0.012 kg/m^2 potash and 0.017 kg/m^2 phosphorus.
